# ConvDeiT-Tiny: Adding Local Inductive Bias to DeiT-Ti for Enhanced Maize Leaf Disease Classification

**DOI:** 10.3390/plants15060982

**Published:** 2026-03-23

**Authors:** Damaris Waema, Waweru Mwangi, Petronilla Muriithi

**Affiliations:** Department of Computing, Jomo Kenyatta University of Agriculture and Technology, Nairobi P.O. BOX 62000-00200, Kenya; waweru_mwangi@icsit.jkuat.ac.ke (W.M.); pmuriithi@jkuat.ac.ke (P.M.)

**Keywords:** ConvDeiT-Tiny, depthwise convolutions, explainable artificial intelligence, CNN-ViT hybrids, maize leaf disease classification, vision transformers, DeiT

## Abstract

Reliable identification of maize leaf diseases is critical for mitigating crop losses, particularly in regions where farmers have limited access to experts. Although vision transformers (ViTs) have recently demonstrated strong performance in image recognition, their weak inductive bias and limited modeling of local texture patterns make them non-ideal for fine-grained maize leaf disease classification. To address these limitations, we propose ConvDeiT-Tiny, a lightweight hybrid ViT that improves DeiT-Ti by placing depthwise convolutions in parallel with multi-head self-attention modules in the first three transformer blocks. The local and global features captured by the convolution and attention modules are concatenated along the embedding dimension and fused using a multilayer perceptron. This results in richer token representations without significantly increasing model size. Across three datasets, ConvDeiT-Tiny (6.9 M parameters) consistently outperformed DeiT-Ti, DeiT-Ti-Distilled, and DeiT-S (21.7 M parameters) when trained from scratch. With transfer learning, ConvDeiT-Tiny achieved an accuracy of 99.15%, 99.35%, and 98.60% on the CD&S, primary, and Kaggle datasets, respectively, surpassing many previous studies with far fewer parameters. For explainability, we present gradient-weighted transformer attribution visualizations showing the disease lesions driving model predictions. These results indicate that injecting local inductive bias in early transformer blocks is beneficial for accurate maize leaf disease classification.

## 1. Introduction

Maize is one of the world’s most widely cultivated cereal crops, serving as a critical source of food, income, and livestock feed, particularly in low- and middle-income countries [1]. However, the productivity of maize farming systems is affected negatively by diseases and pests such as fall armyworms. Traditionally, farmers and extension officers depend on eye inspection for disease and pest identification, which is often time-consuming, labor-intensive, and at times inaccurate [1,2]. These diseases and pests, when left undiagnosed or untreated, can reduce yields by over 50% [3]. Smallholder farmers are particularly more vulnerable to this due to their limited access to diagnostic tools, expert agronomic support, and timely disease management resources. This context underscores the growing need for accurate, automated, affordable, and scalable solutions for in-field disease identification targeting smallholder farmers.

Advancements in deep learning and computer vision have opened up new frontiers in precision agriculture, especially in automating plant disease classification using leaf images [4]. Convolutional neural networks (CNNs) have long dominated this domain due to their strong local feature extraction capabilities [5]. However, the recent success of vision transformers (ViTs) has prompted a paradigm shift. Originally developed for natural language processing (NLP), ViTs adapt the self-attention mechanism to images by dividing them into patches and modeling global dependencies across the image [6]. Unlike CNNs, which rely heavily on inductive biases like locality and translation invariance, ViTs learn relationships more flexibly, making them particularly effective for complex and variable visual patterns common in plant disease symptoms. Although ViTs have established state-of-the-art performance on numerous vision tasks, they are known to under-represent local spatial correlations that convolutional networks capture naturally. Recent hybrid designs that introduce convolutional inductive bias into transformer architectures such as MobileViT [7], CvT [8] and ConVit [9] have shown improved performance, motivating research in hybrid local-global designs for domain-specific tasks.

Several research works have demonstrated the viability of ViTs in identifying complex disease patterns across various crops, yielding promising results, at times outperforming CNN models [1]. For example, Ramadan et al. [1] did a comparative study on maize leaf disease classification using 4 ViTs (ViT-B/16, ViT-B/32, ViT-L/16, and ViT-L/32) and 4 CNNs (DenseNet121, VGG16, ResNet152V2, and InceptionV3). Based on their results, ViT-B/16 achieved the highest accuracy of 94.51% with the Kaggle Corn or Maize Leaf Disease Dataset, outperforming the 4 CNNs and the other ViT variants. In a related study, Miryala and Rasane [10] assessed the performance of ViT and two CNN models (ResNet50 and VGG16) in the classification of sugarcane diseases. They used a sugarcane leaf disease dataset containing 19,926 images. Their results indicated that the ViT model achieved the highest accuracy of 96.53%, beating the two CNN models used.

Even though pure ViTs offer flexibility, scalability, and competitive performance in large-scale computer vision tasks, they present challenges that hinder their application in real-world applications such as crop disease classification. One main limitation is the ViTs’ reliance on large labeled datasets for model training, because it lacks the inherent inductive biases of CNNs, such as locality [11]. As a result, vanilla ViTs are data-hungry and susceptible to overfitting when trained on smaller, domain-specific datasets. These limitations are particularly acute in agricultural domains, where datasets are often small, heterogeneous, and collected under varying environmental conditions with complex backgrounds and varying lighting conditions.

The above challenges have inspired the development and application of CNN-ViT hybrid models in crop disease identification. These models benefit from both the inductive biases of CNNs and the global reasoning of ViTs, achieving better generalization. Shandilya et al. [12] proposed a CNN-ViT hybrid for maize leaf disease classification. In this deep learning model, the CNN module was used to extract local features, whereas the ViT module was used to extract long-range contextual dependencies in the maize leaves through self-attention. The two feature maps obtained from these two models were then concatenated and fed into fully connected layers for classification. This hybrid model achieved an accuracy of 95.93% and 99.06% on the CD&S and the combined Kaggle and Mendeley maize leaf disease datasets, respectively. Another related recent study by Thakur et al. [13] introduced a lightweight hybrid model combining CNNs with a vision transformer for more accurate crop disease classification. The model, consisting of only 0.85 million trainable parameters, achieved impressive performance across five public datasets, including 98.86% accuracy on PlantVillage. Another CNN-ViT Hybrid was proposed by Pang et al. [14] that recorded an accuracy of 99.69% on the CD&S dataset with 20.4 million trainable parameters.

Taken together, the reviewed studies show that complementing the global context captured by ViTs through self-attention with local feature extraction using convolutions improves crop disease classification. Motivated by these findings, we improve DeiT-Ti [15] by proposing ConvDeiT-Tiny, a compact hybrid that places depthwise convolutions into the first three transformer blocks in parallel with multi-head self-attention. The resulting local and global features are concatenated along the token embedding dimension and fused via a 2-layer multilayer perceptron (MLP) with Gaussian Error Linear Unit (GELU) nonlinearity [16]. These architectural choices are motivated by (1) the need to add spatial inductive bias without greatly increasing model size, and (2) evidence from the ablation studies we conducted showing that early-block convolutional injection and non-linear fusion MLP produce the best trade-off between accuracy and efficiency. ConvDeiT-Tiny consistently outperformed both DeiT-Ti and DeiT-Ti-Distilled in the three maize leaf disease datasets used in this paper, including the primary dataset collected by the researchers, when trained from scratch. It also outperformed DeiT-S in all the datasets, despite DeiT-S [15] having more than 3 times the number of trainable parameters in ConvDeiT-Tiny.

Beyond the development of efficient and accurate maize leaf disease classification models, there is a growing need to incorporate Explainable Artificial Intelligence (XAI) techniques to better understand and interpret the decision-making processes of crop disease classification algorithms. These techniques address the issue of interpretability in traditional black-box AI models [17]. Common XAI techniques used in image processing tasks with deep learning include SHapley Additive explanations (SHAP) [18], Local Interpretable Model-agnostic Explanations (LIME) [19], and Gradient-weighted Class Activation Mapping (Grad-CAM) [20]. With the evolution of vision transformers, other XAI techniques that align with ViTs’ internal working have been proposed. These include attention rollout and attention flow [21] as well as the gradient-weighted attention rollout proposed by Chefer et al. [22]. In computer vision tasks, these methods highlight areas in the input image that contributed to the model’s prediction.

Many recent works in crop disease classification have made use of XAI, as it aids in fostering trust among stakeholders such as farmers, agronomists, and policymakers. Authors in [23] implemented Grad-CAM for real-time XAI on an edge device for the classification of grape leaf diseases using a modified MobileNetV3Large. This research obtained a test accuracy of 99.42%. Similar research efforts are reported in [24] where LIME was used to provide explanations for the classification of leaf diseases in vegetables and fruits. For the classification task, this work employed an ensemble of 4 CNNs, namely VGG16, VGG19, ResNet101V2, and InceptionV3, achieving an accuracy of 92.3% across 38 classes using the PlantVillage dataset. Additionally, Özüpak et al. [25] made use of LIME, SHAP, and GRAD-CAM in maize leaf disease classification with a hybrid of MobileNetV2 and ViT. They used the Corn or Maize Leaf Disease Dataset from Kaggle and obtained an accuracy of 96.73%. Peyal et al. [26] also used GRAD-CAM, LIME, and SHAP with a hybrid of CNN and SVM to provide visual explanations through heatmaps, facilitating a clearer understanding of the disease classification process. This work obtained an accuracy of 98.34% in classifying maize leaf diseases in four classes using the PlantVillage dataset obtained from Kaggle.

Overall, existing studies demonstrate that XAI has become essential in crop disease classification. Building on these advancements, the present study incorporates XAI by using gradient-weighted transformer attribution as proposed by Chefer et al. [22] to obtain patch-level relevance maps. These relevance maps highlight the diseased regions, therefore explaining the maize leaf disease classification decisions made by the proposed ConvDeiT-Tiny. By doing this, we also address the concern raised by Khan et al. [11] regarding the opacity of vision transformers’ predictions, and follow their recommendation to incorporate XAI techniques into ViT-based models. Furthermore, this approach allows model predictions to be verified before actions such as the use of fungicides are taken.

In summary, the key contributions of this paper are:

- A lightweight CNN-ViT hybrid architecture that integrates depthwise convolutions into the first three transformer blocks of DeiT-Ti to inject local inductive bias.
- Consistent performance gains across three datasets, surpassing both lightweight and larger ViT models while remaining computationally efficient.
- Extensive ablation studies isolating the benefits of our fusion strategy, token embedding dimension expansion capacity, depthwise convolution placement, and the number of parallel blocks to have.
- Explainability through gradient-weighted attention rollout, showing that ConvDeiT-Tiny attends strongly to relevant disease symptoms for maize leaf disease classification.

Together, these contributions advance the state of the art in maize leaf disease classification by bridging model efficiency, accuracy, and explainability. Experimental validation across three datasets, which also include field-collected images, confirms the effectiveness of ConvDeiT-Tiny, positioning it as a promising solution for digital in-field crop disease management.

## 2. Results

### 2.1. Main Results

The results shown in Table 1 reveal that the proposed ConvDeiT-Tiny consistently outperformed DeiT-Ti, DeiT-Ti-Distilled, and DeiT-S in all three datasets when trained from scratch under identical conditions, achieving an average accuracy of 95.70%. This confirms that complementing self-attention with local feature extraction through depthwise convolutions in the first three transformer blocks improves generalization by improving texture sensitivity. One might argue that ConvDeiT-Tiny performed better than DeiT-Ti (Table 1) because of the increased parameter size. But interestingly, ConvDeiT-Tiny (6.9 M parameters) achieved a higher average accuracy than DeiT-S (21.7 M parameters). This demonstrates that the observed performance gains of ConvDeiT-Tiny originate from architectural enhancements, specifically, the incorporation of local inductive bias in the early layers and an effective feature fusion approach, rather than increased model size.

**Figure 1:**
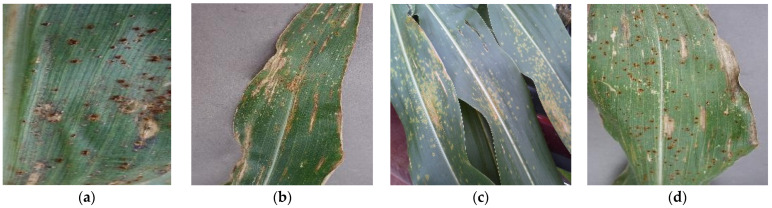
Some of the misclassified images in the COMLDD dataset. (**a**) Actual class is blight while the predicted class is common rust; (**b**) actual class is gray leaf spot while the predicted class is blight; (**c**) actual class is common rust while the predicted class is gray leaf spot; (**d**) actual class is gray leaf spot while the predicted class is blight.

A cross-dataset comparison between ConvDeiT-Tiny and DeiT-Ti shows that these models achieved similar performance trends in the three datasets. They all recorded their highest accuracy with the CD&S dataset, followed by the primary dataset, while the COMLDD dataset recorded the lowest accuracy among these models. The superior performance on the CD&S dataset among these models can be attributed to its relatively clean and well-annotated images. Performance decreased on the field-collected primary dataset, where images contain more background variability and illumination changes. The low accuracy between these two models while using the COMLDD dataset is likely because this dataset contains many images with overlapping disease symptoms, especially among the common rust, blight, and gray leaf spot classes. Images depicting this problem are shown in Figure 1. A closer look at the results presented in Table 1 also reveals that DeiT-S recorded the lowest performance for the primary dataset. This may be explained by its higher model complexity. With substantially more trainable parameters than the other evaluated architectures, DeiT-S (21.7 million trainable parameters) is more prone to overfitting when trained on a relatively small and visually simple dataset. The primary dataset contains three disease categories (healthy, fall armyworm, and northern leaf blight) whose visual symptoms are relatively distinctive, enabling lighter models such as ConvDeiT-Tiny (6.9 million trainable parameters) to learn discriminative features effectively while maintaining better generalization. The fact that ConvDeiT-Tiny outdid DeiT-Ti-Distilled in all the evaluated datasets means that complementing global feature extraction with local feature extraction is more beneficial in domain-specific tasks with small datasets, such as maize leaf disease classification, as opposed to using distillation with a CNN teacher.

Results in Table 1 also show that ConvDeiT-Tiny has more trainable parameters and Multiply-Accumulate Operations (MACs) compared to DeiT-Ti, which is as a result of the added components. The trainable parameters and MACs were recorded with the classification head set to 3 when working with the CD&S dataset.

Results in Table 2 indicate that ConvDeiT-Tiny introduces a moderate increase in training time per epoch and inference latency compared to DeiT-Ti and DeiT-Ti-Distilled. Luckily, this computational overhead is compensated for by its substantially higher classification accuracy across all datasets. Notably, ConvDeiT-Tiny consistently delivers superior classification performance when compared to DeiT-S, despite remaining more lightweight and faster both during training and inference.

After finetuning the pretrained ConvDeiT-Tiny using our three datasets, the classification results on our three evaluation sets were as presented in Table 3. These results show that ConvDeiT-Tiny, with the use of transfer learning, achieves commendable performance, attaining an accuracy of 99.15% for the CD&S dataset, 98.60% for the COMLDD dataset, and 99.35% on our primary, field-collected dataset. These results highlight the importance of leveraging large-scale and diverse datasets to initialize the attention and convolutional components of hybrid transformer models, particularly when working with domain-specific datasets that are often small or have limited intra-class variation. Moreover, the use of TinyImageNet-200 proved to be an effective compromise between full ImageNet-1K pretraining and computational efficiency. These results also reflect the effect of dataset complexity on ConvDeiT-Tiny’s performance. The pretrained ConvDeiT-Tiny achieved the highest accuracy on the primary dataset as this was the simplest of the three datasets because of its easy-to-distinguish classes (healthy, northern leaf blight, and fall armyworm). On the other hand, ConvDeiT-Tiny recorded its lowest accuracy when trained and evaluated with the COMLDD dataset. This is due to confusion caused by overlapping disease symptoms, such as those shown in Figure 1. Despite this, ConvDeiT-Tiny still outperformed all the baseline models in this dataset (COMLDD) as shown in Table 1, exhibiting superior ability to learn complex maize disease patterns.

**Figure 2:**
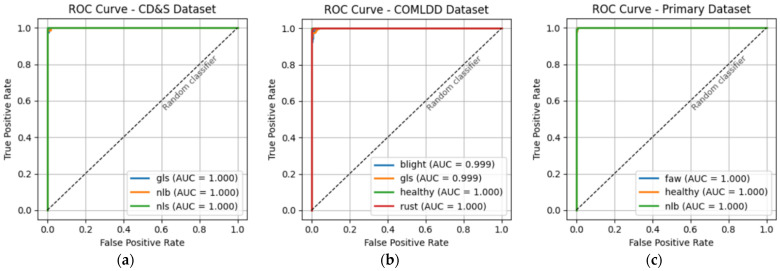
ROC curves after evaluating the pretrained and finetuned ConvDeiT-Tiny with our three datasets. (**a**) CD&S dataset ROC curve; (**b**) COMLDD dataset ROC curve; (**c**) Primary dataset ROC curve.

The receiver operating characteristic (ROC) curves shown in Figure 2 further confirm the excellent discriminative ability of the proposed ConvDeiT-Tiny model across the three evaluated datasets. These ROC curves (Figure 2) show that the area under the curve (AUC) for each disease class was either 0.999 or 1.0, indicating near-perfect separation between diseased and healthy classes over a wide range of decision thresholds. Moreover, the ROC curves nearly approach the top-left corner of the plot for all the datasets, far above the diagonal corresponding to the random classifier, indicating that misclassification errors are largely confined to borderline cases. These ROC-AUC results complement the confusion matrices and accuracy metrics, and provide additional evidence that ConvDeiT-Tiny achieves reliable and consistent performance across diverse maize leaf disease datasets.

**Figure 3:**
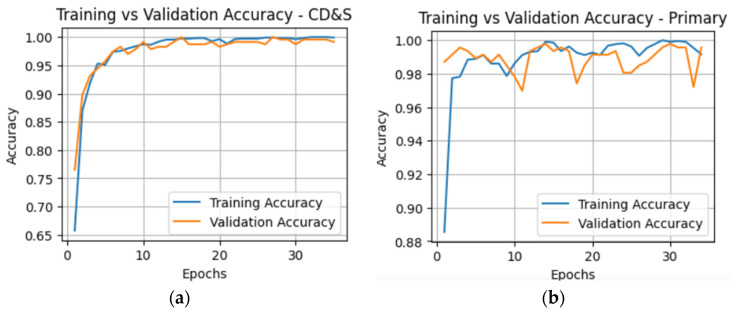
Training and validation accuracy curves for the pretrained ConvDeiT-Tiny. (**a**) CD&S dataset; (**b**) Primary dataset.

The training and validation accuracy curves shown in Figure 3 reveal that the pre-trained ConvDeiT-Tiny converges quickly and shows no significant train-validation gap. This indicates stable learning and good generalization to the validation sets for the primary and CD&S datasets. With the use of early stopping, training for the CD&S dataset stopped at epoch 15, while for the primary dataset, training stopped at epoch 14.

**Figure 4:**
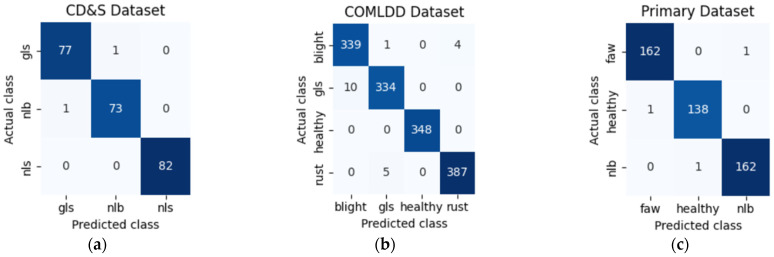
Confusion Matrices after evaluating the pretrained and finetuned ConvDeit-Tiny with our three datasets. (**a**) CD&S dataset confusion matrix; (**b**) COMLDD dataset confusion matrix; (**c**) Primary dataset confusion matrix.

To give a visual representation of ConvDeiT-Tiny’s commendable performance in maize leaf disease classification, we provide three confusion matrices in Figure 4, one for each dataset. These confusion matrices show that the model finetuned and evaluated on the CD&S and primary datasets exhibited near-perfect classification results, with only two and three images, respectively, being misclassified across all test images. On the other hand, twenty images out of one thousand, four hundred and eighty-eight images in the COMLDD were misclassified. This minimal confusion between visually similar disease classes shows the model’s strong discriminative capability, indicating that the proposed parallel block successfully combines local texture awareness with global contextual disease understanding. The fact that misclassifications happened in disease classes with overlapping symptoms such as those shown in Figure 1 suggests that errors were likely due to inherent inter-class similarity rather than model underperformance.

**Figure 5:**
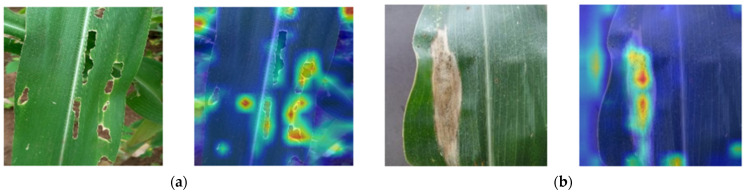
Gradient-weighted transformer attribution explanations for classification decisions made by ConvDeit-Tiny. (**a**) A sample of fall armyworm-infested maize leaf from our primary dataset with its XAI explanation—classification confidence was 100%; (**b**) a sample blight-infested maize leaf from the COMLDD dataset with its XAI explanation—classification confidence was 99.98%.

Finally, we present class-specific transformer attribution visualizations in Figure 5. For the fall armyworm class shown in Figure 5a, the leaf regions exhibiting physical damage and perforations caused by fall armyworm feeding are precisely highlighted. Similarly, for the blight class shown in Figure 5b, the explanation accurately emphasizes the blight regions. By highlighting the areas that positively influenced the model’s decision, these visualizations confirm that the model’s predictions are guided by meaningful disease lesion regions rather than background noise. This reinforces both ConvDeiT-Tiny’s transparency and its practical reliability for real-world crop disease diagnosis.

### 2.2. Results from Ablation Experiments

Results from ablation experiments are presented in Table 4, Table 5, Table 6 and Table 7. These experiments aimed to validate the architectural choices we made for ConvDeiT-Tiny. Specifically, we did ablation studies to find out how many DeiT-Ti blocks to replace with our proposed DWC-MHSA parallel block. These results are presented in Table 4. The results reveal that the highest average accuracy across the three datasets is achieved when only the first three DeiT-Ti blocks are replaced with the proposed DWC-MHSA parallel block. These results indicate that for hybrid vision transformer models, local spatial bias is most beneficial in the early stages, where the extraction of low-level patterns such as edges and texture dominate.

To determine the best placement for depthwise convolutions, we experimented with placing them before MHSA, after MHSA, and in parallel with MHSA. Results in Table 5 depict that the parallel design, albeit having the highest number of parameters, yielded the best accuracy, confirming that local and global features are most complementary when learned jointly rather than sequentially.

To validate the effect of our fusion strategy for the parallel pathways, we assessed it against three other fusion strategies: additive fusion, gated sum fusion, and concatenation followed by a linear layer. The results in Table 6 confirm that a 2-layer fusion MLP with a non-linear mixing function (GELU) enables richer interactions between the heterogeneous spatial (DWC) and global (MHSA) features, improving generalization. Finally, the ablation on the token embedding dimension scaling factor (2C, 4C, and 8C) for the first layer of the fusion MLP was conducted to assess the influence of fusion dimensionality on model performance. Results in Table 7 indicate that 4C achieved the highest accuracy, suggesting that this configuration provides an optimal balance between feature expressiveness and computational efficiency. In contrast, 2C limited the fusion network’s representational capacity, while 8C introduced redundancy that likely degraded performance.

These ablation studies were conducted with the CD&S dataset, apart from the results shown in Table 4, which used all three datasets. Furthermore, apart from the results in Table 4, all the other ablation experiments were performed with the changes made only to the first three transformer blocks.

## 3. Discussion

The goal of this work was to add local inductive bias to DeiT-Ti to improve maize leaf disease classification accuracy. We achieved this by placing depthwise convolutions in parallel with MHSA in the first three transformer blocks. This architectural choice was informed by our ablation experiments, which indicated that replacing the first three transformer blocks (*n* = 3) with the proposed parallel convolution-attention structure yields the best performance, while increasing *n* beyond this value leads to performance degradation. This behavior can be explained by the complementary roles of convolution and self-attention in CNN-ViT hybrids. Convolutional operations introduce strong locality inductive bias, enabling efficient extraction of low-level spatial features in early layers such as edges, disease lesion color, and texture, which are important for accurate maize leaf disease classification. In contrast, deeper transformer layers are more effective at modeling long-range dependencies and global semantic relationships. Prior studies on hybrid vision transformers have similarly reported that introducing convolutional inductive bias in early stages improves optimization and data efficiency, while excessive convolution in deeper layers may limit the global modeling capability of self-attention mechanisms [27]. In a related study, authors in [9] also assert that early vision transformer blocks prefer to learn local features in the input image data than later blocks. Additionally, Xiao et al. [28] also conclude that restricting convolutions to early transformer processing helps strike the balance between the hard inductive biases of CNNs and the global learning capability of vision transformers.

With transfer learning, ConvDeiT-Tiny beat many recent works in maize leaf disease classification, as shown in Table 8. On the CD&S dataset, ConvDeiT-Tiny attained an accuracy of 99.15% with only 6.9 M parameters, surpassing the heavy CNN–ViT hybrid proposed by Shandilya et al. [12] with 87.1 M parameters. However, the DCTN CNN-ViT hybrid proposed by Pang et al. [14] achieved a slightly higher performance than our work (99.69%), but at a cost of an extra 13.5 M parameters. Similarly, on the broader COMLDD dataset, ConvDeiT-Tiny outperformed several CNN-based, pure ViTs, and hybrid architectures, including the work performed by Ramadan et al. [1], despite using far fewer learnable parameters. Among the works that used primary datasets, ConvDeiT-Tiny achieved the highest accuracy of 99.35%, exceeding the accuracy reported in larger models such as in [29,30] for the ViT model. This demonstrates strong generalization capability even on images obtained from the field with challenges like varying lighting conditions and complex, changing backgrounds. Additionally, ConvDeiT-Tiny’s efficiency—accuracy balance highlights its great potential for scalable, real-world deployment, especially in mobile and resource-constrained environments.

## 4. Materials and Methods

### 4.1. The Proposed ConvDeiT-Tiny

#### 4.1.1. The Pipeline of the Proposed ConvDeiT-Tiny

To jointly capture both local and global dependencies in DeiT-Ti, we introduce a parallel block that our proposed architecture uses as the first three blocks of ConvDeiT-Tiny. This parallel block processes tokens through two simultaneous branches: multi-head self-attention (MHSA) and depthwise convolution (DWC), which are placed in parallel. The features from the two branches are then concatenated along the token embedding dimension and fused using a two-layer MLP with GELU activation. The parallel placement avoids the serial bottleneck of MHSA-conv or conv-MHSA used in prior CNN-ViT hybrids such as CvT [8] and CoAtNet [36], and instead allows the model to learn complementary features at the same stage. The intuition follows the parallel-path design philosophy presented in previous studies such as TransConver [37], but with a lighter fusion mechanism tailored for ConvDeiT-Tiny. Recently, Makina et al. [38] also used this parallel extraction of local and global features where a ConvNext model was placed in parallel with the Swin transformer and the hybrid model applied in breast cancer classification. The motivation to replace only the first three DeiT-Ti blocks with our parallel blocks is motivated by recent literature on hybrid ViTs that shows that locality is most beneficial at early stages, where low-level spatial features are extracted [27]. Deeper transformer layers, conversely, benefit from maintaining full global attention without convolutional constraints.

**Figure 6:**
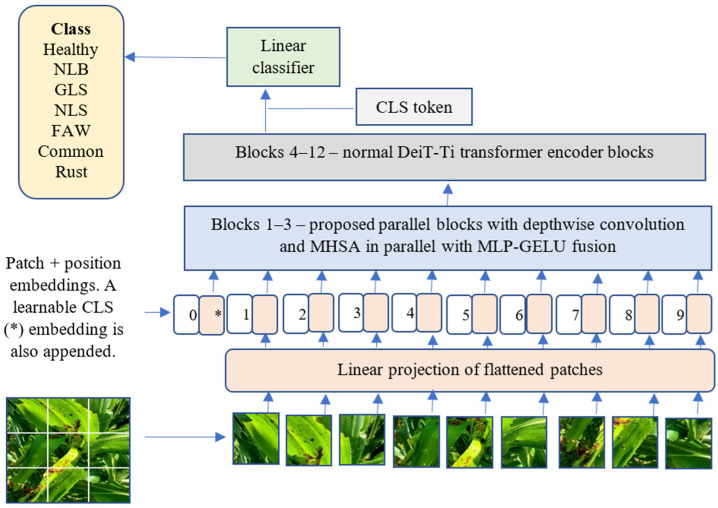
The pipeline of the proposed ConvDeiT-Tiny. * in the sequence of tokens refers to the CLS token. This figure was inspired by Dosovitskiy et al. [6].

**Figure 7:**
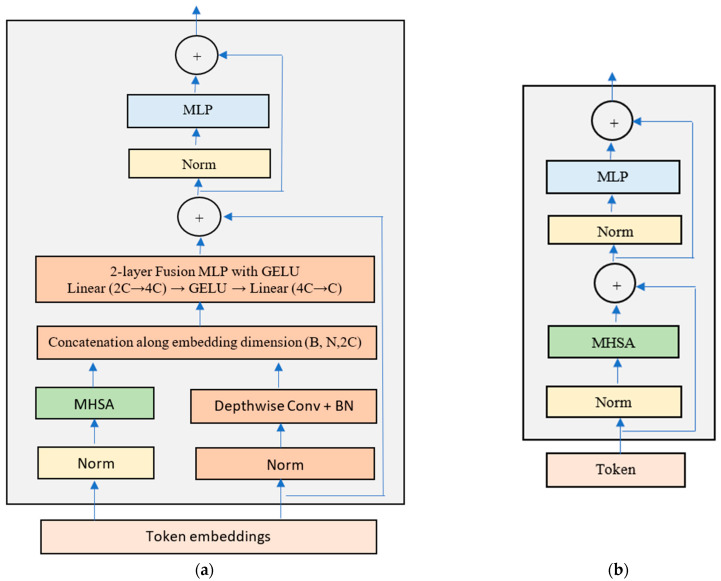
(**a**) The proposed MHSA-depthwise convolution parallel block; (**b**) normal transformer block used in DeiT-Ti. This figure was inspired by a transformer block figure illustrated in [6].

Consistent with DeiT-Ti, ConvDeiT-Tiny employs a single linear classification head operating on the final class token, and therefore, performance gains arise solely as a result of improved feature representations. The pipeline of the proposed ConvDeiT-Tiny is shown in Figure 6. We also present our proposed parallel block in Figure 7a alongside the DeiT-Ti transformer block—Figure 7b.

#### 4.1.2. Justification for Using the Depthwise Convolutions

Depthwise convolutions are a lightweight form of convolution in which a single spatial filter is applied independently to each channel [39]. In contrast to a standard convolution that performs both the channel-wise and spatial-wise computation in one step, a depthwise convolution focuses only on spatial feature extraction, greatly reducing computational cost [40]. According to [39], given a kernel of *D_K_ × D_K_, M* input channels, *N* output channels, and an output feature map of *D_F_ × D_F_*, the computational cost of a standard convolution is given in Equation (1), whereas that of a depthwise convolution is given in Equation (2).

(1) $$Cost_{stdConv}=D_{K}\times D_{K}\times M\times N\times D_{F}\times D_{F}$$

(2) $$Cost_{DWConv}=D_{K}\times D_{K}\times M\times D_{F}\times D_{F}$$

This lightweight nature of depthwise convolutions is what makes them ideal for hybrid transformer architectures like the one proposed in this paper, since they introduce spatial inductive bias without significantly increasing model parameters and computational cost.

#### 4.1.3. Step-by-Step Processing Through the Proposed Parallel Block

i. Input Tokens

The processing of the input image in ConvDeiT-Tiny is similar to how an image is prepared in DeiT-Ti before being passed to the transformer blocks. A 224 × 224 image is first split into 16 × 16 patches, which are linearly projected, and then arranged as a sequence of tokens similar to words in NLP. Positional encoding is also performed to retain the order of the patches. A class (CLS) token is appended to collect information that is finally used for classification. This sequence of tokens is what is given as input to the proposed parallel block and is denoted as $X\in R^{B\times N\times C}$ where *X* is a sequence of token embeddings, and *B* is the batch size. *N* is the number of tokens, and *C* is the token embedding dimension. In simple terms, *X* has the shape *X*: [*Batch*, *197*, *192*].

ii. Normalization per Branch

Layer normalization is applied before the input tokens are fed to both the MHSA and the depthwise convolution branches. We applied the standard formulation shown in Equation (3), which was introduced by Ba et al. [41] and also given by the authors in [42]. In Equation (3), *LN* refers to layer normalization, and *x_i_* is the *ith* feature dimension in the input token. *μ* and *σ* refer to the mean and variance computed across feature dimensions. Finally, *γ* and *β* represent the learnable scale and shift parameters, respectively, which are learned during model training. This is the standard layer normalization equation used in vision transformers, including DeiT-Ti [15].

(3) $$LN\left(x_{i}\right)=\frac{x_{i}-\mu }{\sigma }⊙\gamma +\beta$$

iii. Global Modeling via MHSA

The Multi-Head Self-Attention module forms the core mechanism for global context modeling. It projects each input token into query, key, and value spaces and computes pairwise interactions to capture long-range dependencies among image patches. By dividing the embedding dimension *C* = 192 into three attention heads, the model learns diverse feature subspaces that jointly encode global relational information specific to different maize leaf diseases. The MHSA operation can be summarized in Equation (4), where X is the input token embeddings, *LN*(*X*) is the normalized version of these token embeddings, and *MHSA*(·) applies self-attention to produce new contextualized embeddings. $X_{attn}\in R^{B\times N\times C}$ is the output of the MHSA step, a new set of token embeddings where each token now contains global contextual disease information from all other tokens.

(4) $$X_{attn}=MHSA\left(LN\left(X\right)\right)$$

iv. Local Feature Extraction Via Depthwise Convolutions

In the proposed design, before applying the depthwise convolution, the token sequence is split into two parts: the CLS token (which is not convolved) and patch tokens, which are reshaped into 2-D feature maps before convolution. This means that the input token embeddings are converted from shape $X\in R^{B\times N-1\times C}$ to $X\in R^{B\times C\times H\times W}$ before the convolution operation. This gives the depthwise convolution access to spatial information, which pure attention lacks. The depthwise convolution transformation is defined in Equation (5), where *X* is the input tokens of shape [*B, C, H, W*]. *LN*(·) normalizes *X* before being convolved. DWConv(·) is the depthwise convolution operation that extracts local spatial features in the input patches, with each input channel convolved independently with its own filter (hence no mixing of information across channels). *BN*(·) refers to the batch normalization operation, which is applied after the depthwise convolution to normalize channel-wise activations to stabilize training and improve generalization. Batch normalization is especially helpful before fusion, as it causes the depthwise convolution pathway to produce stable and comparable feature scales when fused with the MHSA pathway. Lastly, *X_DWConv_* represents the local-aware patch embeddings, which are the final output of the depthwise convolution module.

(5) $$X_{DWConv}=BN\left(DWConv\left(LN\left(X\right)\right)\right)$$

The output of the depthwise convolution operation is $X_{DWConv}\in R^{B\times C\times H\times W}$. This is then converted back to a tensor of shape $X_{DWConv}\in R^{B\times N-1\times C}$ after which the Cls token of shape $Cls\in R^{B\times 1\times C}$ is appended back to give $X_{DWConv}\in R^{B\times N\times C}$. This tensor shape conversion is important as it converts the tensor back to the shape similar to the output shape of the MHSA, which is necessary for feature fusion.

In summary, this depthwise convolution branch captures short-range visual structures to complement the long-range global dependencies modeled by attention.

v. Feature Concatenation

After obtaining global contextual features from the MHSA branch, $X_{attn}\in R^{B\times N\times C}$ and local spatial features from the depthwise convolution branch, $X_{DWConv}\in R^{B\times N\times C}$, the two representations are concatenated along the token embedding dimension. This results in *X_concat_*, formally defined as $X_{concat}\in R^{B\times N\times 2C}$, with a combined token embedding of size 2C (384). The concatenation step is shown in Equation (6).

(6) $$X_{concat}=\left[X_{attn}\right\|X_{DWConv}]$$

vi. Feature Fusion Using a 2-Layer Fusion MLP with GELU

To enable effective cross-branch interaction, we employ a two-layer fusion MLP. The first linear layer expands the token embeddings of the concatenated features from 2C (384) to 4C (768). This is followed by the GELU activation function [16] to allow non-linear feature mixing. Finally, the second MLP linear layer compresses the token embeddings from 4C back to C, in line with the tensor shape expected by the next transformer components. Formally, the fused representation is computed as in Equation (7), where *W*_1_ and *W*_2_ are the weight matrices for the first and second linear layer in the MLP fusion, and ϕ(·) is the GELU activation function. $X_{fused}\in R^{B\times N\times C}$ is the final fused token representation, where local and global information is jointly encoded before the next residual connection and MLP operation.

(7) $$X_{fused}=W_{2(4C\to C)}\phi \left(W_{1(2C\to 4C}\right)(X_{concat}))$$

vii. Residual Refinement Through the Transformer MLP

Following feature fusion, the fused representation *X_fused_* is first added to the original input tokens *X*, forming a residual pre-activation *X + X_fused_*. This intermediate representation is normalized and passed through the transformer MLP to enhance token-wise feature interactions. The final output is obtained through a single residual shortcut, expressed in Equation (8). This refinement step preserves the original information flow, strengthens feature expressiveness, and maintains training stability. $X_{out}\in R^{B\times N\times C}$ is the final parallel block output passed down to the next block.

(8) $$X_{out}=(X+X_{fused})+MLP(LN(X+X_{fused}))$$

From the foregoing discussion, it is clear that the proposed parallel block is shape-preserving: given an input tensor $X\in R^{B\times N\times C}$, the output $X_{out}\in R^{B\times N\times C}$ retains the same dimensionality, ensuring compatibility with subsequent standard transformer pipelines. Hence, *X* = *X_out_* = [*B*, *197*, *192*].

### 4.2. Experiments

#### 4.2.1. Data Source and Preparation

Our research used three maize leaf disease datasets: the Corn Disease and Severity (CD&S) dataset [43], the Corn or Maize Leaf Disease Dataset available on Kaggle (henceforth abbreviated as COMLDD) [44], and lastly, primary data we collected for our research. The CD&S dataset had a total of 1571 images across three disease classes: gray leaf spot—GLS (523), northern leaf blight—NLB (497), and northern leaf spot—NLS (551). Images in every class were split randomly into 70% for training, 15% for validation, and 15% for testing. On the other hand, the COMLDD dataset had a total of 4188 images across four classes: GLS (574), NLB (1146), common rust (1306), and healthy (1162). Images in each class in the COMLDD dataset were randomly split into 70% for training, 15% for the validation set, and 15% for the test set. After data splitting, we performed data augmentation for every class in all dataset partitions in the COMLDD to artificially increase the dataset size and variety. Specifically, images in the healthy, NLB, and common rust classes underwent horizontal flipping. On the other hand, images in the GLS class underwent vertical flipping, 90-degree clockwise rotation, and a vertical flip for the rotated images to reduce class imbalance. After these, the total number of images in each class was NLB (2292), GLS (2296), common rust (2612), and healthy (2324). We performed offline data augmentation only for this dataset before model training because many images in this dataset have overlapping disease symptoms, and we needed to provide enough samples for training, validation, and evaluation of ConvDeiT-Tiny and our baseline models. Table 9 shows the number of images per disease class in the COMLDD before and after augmentation.

The primary data used in this work was collected by the researchers between the year 2023 and 2025. This data was collected from the Jomo Kenyatta University of Agriculture and Technology (JKUAT) farm, maize farms in the western regions of Kenya, and the SAJOREC farm situated in JKUAT. The data was collected using mobile phones and a tablet, devices that are very common in automated crop disease identification. During data collection, we ensured that the dataset captured real challenges found in field images, like varying backgrounds, different illumination conditions, occlusion, and noise like dust, tassels, and raindrops. With the help of a maize leaf disease specialist, we categorized the collected data into three classes: healthy, northern leaf blight, and fall armyworm. This categorization was guided by biological symptoms on the collected leaf images. All the images with uniform green color on the captured maize leaves went to the healthy class. In addition, images showing fall armyworm perforations, perforations together with the fall armyworm pest, and perforations together with fall armyworm waste were put in the fall armyworm class. Lastly, the images that were put in the northern leaf blight class were those with relevant symptoms such as long, elliptical gray-green to tan lesions, ranging from 2.5 cm to 15 cm in length along the leaf veins. The total number of images in this dataset was 3089, distributed as follows: healthy (925), NLB (1082), and fall armyworm (1082). Like the other two datasets, these images were also randomly split into 70% for training, 15% for validation, and 15% for testing.

Before being used for model training and evaluation, the images in the three datasets were resized to 224 × 224, as this is the required input size in ConvDeiT-Tiny (just as in DeiT-Ti). The image data was also normalized before the experiments, as image data normalization has been found to improve deep learning outcomes by removing the impact of irrelevant pixel variations, causing the model to focus on learning only important features [45]. Additionally, we added Gaussian noise, Gaussian blur, and color jitter to a random 20% of the training and validation data during data loading (online augmentation), where one of the three transformations was randomly selected. For Gaussian noise, we used a mean of 0 and a standard deviation of 0.05, while a Gaussian blur was applied using a radius of 1 pixel. Lastly, color jitter was performed with brightness being set to 0.2, contrast = 0.2, saturation = 0.2, and hue = 0.1. These three types of noise simulate realistic challenges present in field-acquired images, and exposing the model to them during training makes ConvDeiT-Tiny more resilient to real-world noise and color inconsistencies, which are common in practical deployment scenarios [46]. Other than noise addition, we also performed random online data augmentation through random vertical flipping and random rotation within 0–270 degrees for the training sets across the three datasets. Online data augmentation means that transformations are applied dynamically during model training rather than creating additional stored images. This improves model generalization by exposing the model to different variations and orientations of the same image across training epochs.

#### 4.2.2. Ablation Studies

For us to justify the final architectural choices for ConvDeiT-Tiny, we conducted a series of ablation experiments. Each study isolated a single design factor while keeping all other components unchanged. The results for these ablation studies are in Section 3 of this paper. The following ablation experiments were carried out:

- Number of parallel blocks—for us to find out which blocks benefited the most from complementary local feature extraction, we experimented with replacing between 1 and 12 Transformer blocks with our proposed depthwise convolution-MHSA parallel block.
- Placement of depthwise convolutions in relation to the MHSA module—we assessed placing the depthwise convolution before MHSA, after MHSA, and in parallel with MHSA.
- Token embedding dimension expansion capacity for the MLP fusion module—we experimented with an embedding scaling factor of 2, 4, and 8.
- Fusion strategy—in addition to the MLP-GELU fusion used in the proposed ConvDeiT-Tiny, we experimented with concatenation followed by a linear layer, additive fusion, and gated sum fusion.

#### 4.2.3. Baseline Models

To verify the effect of adding depthwise convolutions in parallel with MHSA for improving maize leaf disease classification, we assess ConvDeiT-Tiny against three DeiT vision transformers: DeiT-Ti, DeiT-Ti-Distilled, and DeiT-S [15]. DeiT-Ti is used as a benchmark because it is the architecture our work modifies. DeiT-Ti-Distilled is used to show whether using distillation from a CNN teacher is better than adding depthwise convolutions as proposed in our work. Lastly, DeiT-S (21.7 M trainable parameters) is used for parameter-fairness assessment, as it helps to verify that ConvDeiT-Tiny (6.9 M trainable parameters) performed better than DeiT-Ti primarily as a result of injecting local inductive bias and not merely from an increased parameter count.

#### 4.2.4. Model Training

All experiments reported in this paper were conducted using the P5000 GPU machine, made available through a Paperspace Gradient subscription. The machine had 30 GB RAM, 8 CPUs, and 16 GB of GPU memory. ConvDeiT-Tiny and the baseline models (DeiT-Ti, DeiT-Ti-Distilled, and DeiT-S) were implemented using PyTorch 2.7.1, Python 3.11.7, CUDA 12.6, and timm 1.0.15 on Ubuntu 22.04.3 LTS. For reproducibility, we used a constant seed of 42 in all model training and evaluation experiments.

For ablation studies and all the experiments whose goal was to compare the performance of ConvDeiT-Tiny and the baseline vision transformers, we opted to train the models from scratch as opposed to using ImageNet-pretrained weights. This choice ensured a fair comparison by isolating the impact of architectural changes from the benefits of ImageNet pretraining. We optimized all models using the Cross-Entropy loss function, which is widely used for multi-class image classification. For from-scratch model training, we used Adam as the optimizer, a batch size of 32, a learning rate of 0.0001, weight decay of 0, and no scheduler. We kept these hyperparameters constant during ablation experiments. This ensures that performance differences arise only from architectural variations, making ablation analysis reliable. To prevent overfitting during model training, we used early stopping with 200 epochs and a patience value of 20. The final trained model was selected based on its validation accuracy.

For us to ensure a fair comparison with prior work on maize leaf disease classification, which predominantly relies on transfer learning in addition to architectural changes, we adopted a hybrid pretraining strategy for ConvDeiT-Tiny. To be specific, all the unchanged DeiT-Ti components retained their ImageNet-1K pretrained weights, whereas the newly introduced convolutional components and fusion MLP blocks were pretrained on the Tiny-ImageNet-200 [47] dataset with all other DeiT-Ti components frozen. This hybrid approach preserves the strong global features learned from the large-scale natural Imagenet-1K dataset while allowing the modified local feature pathways and fusion MLP blocks to acquire meaningful representations before fine-tuning. Tiny-ImageNet-200 was selected due to its class diversity, closer scale to ImageNet-1K, and its suitability for lightweight pretraining without excessive compute requirements. For this pretraining step, we also used the Cross-Entropy loss function, Adam, a batch size of 32, a learning rate of 0.0001, weight decay of 0, and no scheduler. Furthermore, we made use of early stopping with 200 epochs and a patience of 20.

With the weights obtained after pretraining, we finetuned ConvDeiT-Tiny on our three datasets. To ensure that we obtained the best possible models, we performed a grid search for the primary and CD&S datasets using the following options: optimizer: Adam, AdamW; learning rate: 0.00001, 0.0001; weight decay: 0.001, 0; learning rate scheduler: cosine annealing, none. During the grid search, a constant batch size of 32 was used. We also made use of early stopping with a patience of 20 to prevent overfitting and saved the best weights based on validation accuracy. Since there are overlapping disease classes in the CD&S and COMLDD datasets, we did not perform a grid search for the COMLDD dataset. Instead, we used the same parameters obtained after running a grid search for the CD&S dataset to finetune ConvDeiT-Tiny on the COMLDD dataset. Table 10 shows the best hyperparameters for each dataset based on the grid search.

#### 4.2.5. Model Evaluation

To assess the performance of ConvDeiT-Tiny, we used accuracy, precision, recall, and F1-Score as the main metrics. Accuracy measures the proportion of total correct predictions, and its computation is shown in Equation (9). Precision measures the percentage of instances that the model classified as positive that were actually positive, and its computation is shown in Equation (10). On the other hand, recall, whose computation is shown in Equation (11), answers the question: out of all actual positives, how many did the model correctly predict? Finally, F1-score, shown in Equation (12), is the harmonic mean of precision and recall. In Equations (9)–(12), *TP*, *TN*, *FP*, and *FN* refer to true positive, true negative, false positive, and false negative, respectively.

(9) $$Accuracy=\frac{TP+TN}{TP+TN+FP+FN}$$

(10) $$Precision=\frac{TP}{TP+FP}$$

(11) $$Recall=\frac{TP}{TP+FN}$$

(12) $$F1\text{-}Score=2\times \frac{Precision\times Recall}{Precision+Recall}$$

In addition to the above metrics, we used confusion matrices to visualize and analyze the model’s classification performance across all classes. Furthermore, we used ROC-AUC curves to complement standard classification metrics like accuracy and recall. Additionally, gradient-weighted transformer attribution was used to provide interpretability by highlighting the lesions that most influenced the model’s predictions for individual samples. This helped in confirming that the model was actually focusing on relevant disease areas to classify a maize leaf image into a particular disease class.

When evaluating the effect of the architectural changes introduced by ConvDeiT-Tiny on inference time, we measured the forward-pass latency of each model under a controlled setup. All inference experiments were performed under the same software and hardware configurations using 234 images from the CD&S dataset’s test set. Before measurement, we executed 30 warm-up batches to eliminate the overhead associated with CUDA kernel initialization, cuDNN autotuning, and to allow the GPU to reach a stable operating frequency. During timing, we evaluated the model only, excluding preprocessing, data loading, and I/O operations, to obtain a reliable estimate of model execution time. We repeated each inference measurement for 5 runs to reduce timing noise and obtain statistically stable latency measurements. From this 5-run distribution of per-image latencies, we recorded the median value as our inference time for one image. Based on this median value, we computed the models’ throughput (number of images per second). This setup provides a fair and hardware-consistent comparison, isolating the impact of the proposed architectural changes on inference time.

## 5. Conclusions

We introduce ConvDeiT-Tiny, an enhanced variant of DeiT-Ti that injects local inductive bias into the first three transformer blocks through a parallel depthwise convolution-MHSA design. These features are then concatenated along the token embedding dimension and fused using a two-layer fusion MLP with GELU activation. By complementing DeiT-Ti’s global representations with local feature extraction, the proposed architecture achieved superior performance on three maize leaf disease datasets while maintaining a lightweight footprint. The gradient-weighted transformer attribution visualizations provided show that ConvDeiT-Tiny actually focuses on relevant disease lesions for classification, fostering trust among stakeholders in the prediction decisions made by the model. Although ConvDeiT-Tiny incurs higher training and inference times compared to DeiT-Ti and DeiT-Ti-Distilled, the performance gains justify this overhead. Additionally, the computational cost of our proposed architecture remains well below that of DeiT-S, while being more accurate.

While ConvDeiT-Tiny performs better in maize leaf disease classification than DeiT-Ti, DeiT-Ti-Distilled, and DeiT-S under similar training conditions, there are several opportunities for future improvement. First, future work can focus on compressing and optimizing the model to make it run on low-end mobile phones and edge devices, enabling offline disease diagnosis in remote farming regions where internet connectivity is limited. Second, we recommend end-to-end pretraining on the full ImageNet-1K dataset, which may further improve generalization and unlock additional performance gains beyond Tiny-ImageNet-200 pre-training. Third, broader validation across different crop species and non-agricultural domains would help test the robustness of the proposed architecture and confirm its applicability beyond maize leaf disease classification.

In summary, ConvDeiT-Tiny advances lightweight CNN-ViT hybrid designs for maize leaf disease classification and demonstrates promising real-world applicability given its high performance even on field-collected images.

## Figures and Tables

**Table 1:** Results showing how ConvDeiT-Tiny performed in terms of accuracy in comparison to DeiT-Ti, DeiT-Ti-Distilled, and DeiT-S after being trained from scratch with our three maize leaf disease datasets.

Model	Trainable Parameters	MACs	CD&S	COMLDD	Primary	Average
DeiT-Ti	5,524,995	913,640,583	95.73%	93.14%	94.19%	94.35%
DeiT-Ti-Distilled	5,525,958	919,051,542	90.17%	92.51%	94.19%	92.29%
DeiT-S	21,666,819	3,221,625,099	95.30%	94.54%	92.69%	94.18%
ConvDeiT-Tiny	6,864,195	1,178,023,431	97.01%	95.03%	95.05%	95.70%

**Table 2:** Effects of ConvDeiT-Tiny on training and inference time. Training is done from scratch. The CD&S dataset is used for both model training and evaluation.

Model	Training Time Per Epoch (Seconds)	Total Training Time (Seconds)	Inference Time Per Image (Milliseconds)	Inference Throughput (Images/Second)	Test Accuracy
DeiT-Ti	5.44	358.77	0.889	1124.86	95.73%
DeiT-Ti-Distilled	5.48	279.24	0.894	1118.57	90.17%
DeiT-S	11.04	1037.43	2.530	395.26	95.30%
ConvDeiT-Tiny	7.07	671.27	1.066	938.01	97.01%

**Table 3:** Results showing the performance of the pretrained ConvDeiT-Tiny on maize leaf disease classification.

Dataset	Accuracy	Precision	Recall	F1-Score	Macro-Average AUC
CD&S dataset	99.15%	99.12%	99.12%	99.12%	0.9999
COMLDD dataset	98.60%	98.59%	98.59%	98.59%	0.9996
Primary dataset	99.35%	99.35%	99.35%	99.35%	1.0000

**Table 4:** Ablation results on the number of DeiT-Ti blocks to replace with the proposed DWC-MHSA parallel block.

No. of Parallel Blocks	No. of Trainable Parameters	CD&S Dataset	COMLDD Dataset	Primary Dataset	Average Accuracy
*n* = 1	5,971,395	95.73%	93.98%	93.55%	94.42%
*n* = 2	6,417,795	96.15%	95.03%	93.98%	95.05%
*n* = 3	6,864,195	97.01%	95.03%	95.05%	95.70%
*n* = 4	7,310,595	95.73%	95.80%	95.48%	95.67%
*n* = 5	7,756,995	96.15%	93.07%	92.69%	93.96%
*n* = 6	8,203,395	94.44%	93.91%	95.48%	94.61%
*n* = 7	8,649,795	95.73%	94.40%	95.27%	95.13%
*n* = 8	9,096,195	94.02%	94.05%	95.27%	94.45%
*n* = 9	9,542,595	95.73%	94.82%	94.84%	95.13%
*n* = 10	9,988,995	92.74%	95.17%	95.27%	94.39%
*n* = 11	10,435,395	95.73%	94.89%	95.27%	95.30%
*n* = 12	10,881,795	94.87%	95.66%	92.69%	94.41%

**Table 5:** Ablation results on different placements of the depthwise convolutions.

Depthwise Convolution Placement	No. of Trainable Parameters	Test Accuracy
DWC after MHSA	5,531,907	88.03%
DWC before MHSA	5,533,059	92.31%
DWC in parallel with MHSA	6,864,195	97.01%

**Table 6:** Ablation results on different fusion techniques.

Fusion Strategy	No. of Trainable Parameters	Test Accuracy
Concatenation + linear layer	5,755,971	95.30%
Additive fusion	5,645,379	93.56%
Gated sum fusion	5,534,214	92.31%
Fusion MLP with GELU	6,864,195	97.01%

**Table 7:** Ablation results on fusion MLP token embedding dimension expansion capacity.

Embedding Expansion Capacity	No. of Trainable Parameters	Test Accuracy
C = 2	6,199,491	96.58%
C = 4	6,864,195	97.01%
C = 8	8,193,603	94.87%

**Table 8:** How the results of ConvDeiT-Tiny compare with those of previous similar works in maize leaf disease classification.

Authors	Algorithms Used	Dataset	No. of Parameters	Reported Accuracy
[31]	DFCANet (Double Fusion block with Coordinate Attention Network)	CD&S	1.91 M	98.47%
[12]	CNN-Vit hybrid	CD&S	87 M	95.93%
[14]	DCTN-CNN-ViT Hybrid	CD&S	20.4 M	99.69%
Ours	ConvDeiT-Tiny	CD&S	6.9 M	99.15%
[32]	ResNet152	COMLDD	60 M	98.34%
[33]	A lightweight CNN	COMLDD	1.22 M	94.97%
[34]	Hybrid Adaptive Swarm-enhanced Vision Transformer (HAS-ViT)	COMLDD	N/A	98.1%
[1]	Assessed 8 models DenseNet121, VGG16, ResNet152V2, InceptionV3, ViT-B/16, ViT-B/32, ViT-L/16, ViT-L/32	COMLDD	86 M	94.51 (ViT-B/16)
[25]	MobileNetV2 and Vision Transformer (ViT) stacking model.	COMLDD	N/A	96.73%
Ours	ConvDeiT-Tiny	COMLDD	6.9 M	98.60%
[29]	ResNet50	Primary	25.6 M	98.18%
[30]	ViT	Primary plus web obtained images	86.9 M	90%
[30]	State-space attention mechanism combined with a multi-scale feature fusion	Primary plus web obtained images	N/A	94%
[35]	A stacking ensemble of 4 CNNs (DenseNet201, InceptionNetV3, NASNetMobile, VGG19) and Vision Transformer	Primary data plus Kaggle dataset	N/A	99.15%
Ours	ConvDeiT-Tiny	Primary	6.9 M	99.35%

**Table 9:** The number of images per class for the COMLDD before and after augmentation.

Disease Class	Training Set (Original)	Training Set (Augmented)	Validation Set (Original)	Validation Set (Augmented)	Test Set (Original)	Test Set (Augmented)
Blight	802	1604	172	344	172	344
Gray leaf spot	402	1608	86	344	86	344
Common rust	914	1828	196	392	196	392
Healthy	814	1628	174	348	174	348

**Table 10:** The best hyperparameters for each dataset as obtained through a grid search during model finetuning.

Dataset	Optimizer	Learning Rate	Weight Decay	Scheduler
CD&S dataset	Adam	0.00001	0.001	Cosine annealing
COMLDD dataset	Adam	0.00001	0.001	Cosine annealing
Primary dataset	Adam	0.0001	0	Cosine annealing

## Data Availability

The program code and dataset splits for the three datasets used in this study, including our primary data, can be found at https://github.com/DamarisWaema/ConvDeiT-Tiny (accessed on 18 March 2026).

## Abbreviations

The following abbreviations are used in this manuscript:

ViT	Vision transformer
MLP	Multilayer perceptron
GELU	Gaussian Error Linear Unit
CNNs	Convolutional neural networks
AI	Artificial intelligence
XAI	Explainable artificial intelligence
LIME	Local Interpretable Model-agnostic Explanation
SHAP	SHapley Additive explanations
Grad-CAM	Gradient-weighted Class Activation Mapping
DWC	Depthwise Convolution
MHSA	Multi-head self-attention
DeiT	Data-Efficient Image Transformer
CD&S	Corn Disease and Severity dataset
COMLDD	Corn or maize leaf disease dataset
ROC	Receiver operating characteristic
AUC	Area under the curve

## References

[1] Ramadan S.T.Y., Sakib T., Jahangir R., Rahman S., Bhattacharyya S., Banerjee J.S., Köppen M. (2024). Maize Leaf Disease Detection Using Vision Transformers (ViTs) and CNN-Based Classifiers: Comparative Analysis. Human-Centric Smart Computing.

[2] Zhou H., Su Y., Chen J., Li J., Ma L., Liu X., Lu S., Wu Q. (2024). Maize Leaf Disease Recognition Based on Improved Convolutional Neural Network ShuffleNetV2. Plants.

[3] Amin H., Darwish A., Hassanien A.E., Soliman M. (2022). End-to-End Deep Learning Model for Corn Leaf Disease Classification. IEEE Access.

[4] Pacal I., Işık G. (2025). Utilizing Convolutional Neural Networks and Vision Transformers for Precise Corn Leaf Disease Identification. Neural Comput. Appl..

[5] Maurya R., Mahapatra S., Rajput L. (2024). A Lightweight Meta-Ensemble Approach for Plant Disease Detection Suitable for IoT-Based Environments. IEEE Access.

[6] Dosovitskiy A., Beyer L., Kolesnikov A., Weissenborn D., Zhai X., Unterthiner T., Dehghani M., Minderer M., Heigold G., Gelly S. (2020). An Image Is Worth 16x16 Words: Transformers for Image Recognition at Scale. arXiv.

[7] Wadekar S.N., Chaurasia A. (2022). MobileViTv3: Mobile-Friendly Vision Transformer with Simple and Effective Fusion of Local, Global and Input Features. arXiv.

[8] Wu H., Xiao B., Codella N., Liu M., Dai X., Yuan L., Zhang L. CvT: Introducing Convolutions to Vision Transformers. Proceedings of the 2021 IEEE/CVF International Conference on Computer Vision (ICCV).

[9] d’Ascoli S., Touvron H., Leavitt M.L., Morcos A.S., Biroli G., Sagun L. ConViT: Improving Vision Transformers with Soft Convolutional Inductive Biases. Proceedings of the 38th International Conference on Machine Learning.

[10] Miryala S., Rasane K. (2025). Enhancing Sugarcane Leaf Disease Classification Using Vision Transformers over CNNs. Discov. Artif. Intell..

[11] Khan S., Naseer M., Hayat M., Zamir S.W., Khan F.S., Shah M. (2022). Transformers in Vision: A Survey. ACM Comput. Surv..

[12] Shandilya G., Gupta S., Mohamed H.G., Bharany S., Rehman A.U., Hussen S. (2025). Enhanced Maize Leaf Disease Detection and Classification Using an Integrated CNN-ViT Model. Food Sci. Nutr..

[13] Thakur P.S., Chaturvedi S., Khanna P., Sheorey T., Ojha A. (2023). Vision Transformer Meets Convolutional Neural Network for Plant DiseaseClassification. Ecol. Inform..

[14] Pang D., Wang H., Ma J., Liang D. (2023). DCTN: A Dense Parallel Network Combining CNN and Transformer for Identifying Plant Disease in Field. Soft Comput..

[15] Touvron H., Cord M., Douze M., Massa F., Sablayrolles A., Jégou H. Training Data-Efficient Image Transformers & Distillation through Attention. Proceedings of the 38th International Conference on Machine Learning.

[16] Hendrycks D., Gimpel K. (2016). Gaussian Error Linear Units (GELUs). arXiv.

[17] Mulwa M.M., Mwangi R.W., Mindila A. (2024). GMM-LIME Explainable Machine Learning Model for Interpreting Sensor-Based Human Gait. Eng. Rep..

[18] Lundberg S.M., Lee S.-I. (2017). A Unified Approach to Interpreting Model Predictions. Adv. Neural Inf. Process. Syst..

[19] Ribeiro M.T., Singh S., Guestrin C. “Why Should I Trust You?”: Explaining the Predictions of Any Classifier. Proceedings of the ACM SIGKDD International Conference on Knowledge Discovery and Data Mining.

[20] Selvaraju R.R., Cogswell M., Das A., Vedantam R., Parikh D., Batra D. Grad-CAM: Visual Explanations from Deep Networks via Gradient-Based Localization. Proceedings of the IEEE International Conference on Computer Vision.

[21] Abnar S., Zuidema W. Quantifying Attention Flow in Transformers. Proceedings of the 58th Annual Meeting of the Association for Computational Linguistics.

[22] Chefer H., Gur S., Wolf L. Transformer Interpretability Beyond Attention Visualization. Proceedings of the 2021 IEEE/CVF Conference on Computer Vision and Pattern Recognition (CVPR).

[23] Karim M.J., Goni M.O.F., Nahiduzzaman M., Ahsan M., Haider J., Kowalski M. (2024). Enhancing Agriculture through Real-Time Grape Leaf Disease Classification via an Edge Device with a Lightweight CNN Architecture and Grad-CAM. Sci. Rep..

[24] Ammar O.A.D., Abbas S.S., Zafar A., Akram B.A., Dong F., Talpur M.S.H., Uddin M. (2024). Plant Leaf Disease Detection Using Ensemble Learning and Explainable AI. IEEE Access.

[25] Özüpak Y., Alpsalaz F., Aslan E., Uzel H. (2025). Hybrid Deep Learning Model for Maize Leaf Disease Classification with Explainable AI. N. Z. J. Crop Hortic. Sci..

[26] Peyal H.I., Mondal N.I.M., Miraz S. (2024). An Efficient Explainable AI Method Combining CNN and SVM for Corn Leaf Disease Detection and Visualization. 2024 27th International Conference on Computer and Information Technology (ICCIT), Cox’s Bazar, Bangladesh, 20–22 December 2024.

[27] Li Y., Zhang K., Cao J., Timofte R., Magno M., Benini L., Van Goo L. LocalViT: Analyzing Locality in Vision Transformers. Proceedings of the IEEE International Conference on Intelligent Robots and Systems.

[28] Xiao T., Singh M., Mintun E., Darrell T., Dollár P., Girshick R. Early Convolutions Help Transformers See Better. Proceedings of the 35th Conference on Neural Information Processing Systems (NeurIPS 2021).

[29] Micheni M., Kinyua M., Too B., Gakii C. (2021). Maize Leaf Disease Detection Using Convolutional Neural Networks. J. Appl. Comput. Sci. Math..

[30] Zhu T., Yan F., Lv X., Zhao H., Wang Z., Dong K., Fu Z., Jia R., Lv C. (2024). A Deep Learning Model for Accurate Maize Disease Detection Based on State-Space Attention and Feature Fusion. Plants.

[31] Chen Y., Chen X., Lin J., Pan R., Cao T., Cai J., Yu D., Cernava T., Zhang X. (2022). DFCANet: A Novel Lightweight Convolutional Neural Network Model for Corn Disease Identification. Agriculture.

[32] Gopalan K., Srinivasan S., Pragya P., Singh M., Mathivanan S.K., Moorthy U. (2025). Corn Leaf Disease Diagnosis: Enhancing Accuracy with Resnet152 and Grad-Cam for Explainable AI. BMC Plant Biol..

[33] Alpsalaz F., Özüpak Y., Aslan E., Uzel H. (2025). Classification of Maize Leaf Diseases with Deep Learning: Performance Evaluation of the Proposed Model and Use of Explicable Artificial Intelligence. Chemom. Intell. Lab. Syst..

[34] Patil N.S., Kannan E. (2026). Hybrid Adaptive Swarm Enhanced Vision Transformer for Accurate Corn Leaf Disease Prediction. Fusion Pract. Appl..

[35] Weldeslasie D.T., Mekonen M.Y., Abebe A.M., Desta K.T. (2025). A Statistically Validated Stacking Ensemble of CNNs and Vision Transformer for Robust Maize Disease Classification. Discov. Artif. Intell..

[36] Dai Z., Liu H., Le Q.V., Tan M. (2021). CoAtNet: Marrying Convolution and Attention for All Data Sizes. Adv. Neural Inf. Process. Syst..

[37] Liang J., Yang C., Zeng M., Wang X. (2022). TransConver: Transformer and Convolution Parallel Network for Developing Automatic Brain Tumor Segmentation in MRI Images. Quant. Imaging Med. Surg..

[38] Makina M., Chacha W.M., Mwangi R.W. (2026). A Hybrid SwinTConvNeXt with Learnable Dynamic Gating Fusion for Breast Cancer Histopathology Image Classification. Eng. Rep..

[39] Howard A.G., Zhu M., Chen B., Kalenichenko D., Wang W., Weyand T., Andreetto M., Adam H. (2017). MobileNets: Efficient Convolutional Neural Networks for Mobile Vision Applications. arXiv.

[40] Guo Y., Li Y., Wang L., Rosing T. (2019). Depthwise Convolution Is All You Need for Learning Multiple Visual Domains. Proc. AAAI Conf. Artif. Intell..

[41] Ba J.L., Kiros J.R., Hinton G.E. (2016). Layer Normalization. arXiv.

[42] Gupta A., Ozdemir A., Anumanchipalli G. (2025). Geometric Interpretation of Layer Normalization and a Comparative Analysis with RMSNorm. arXiv.

[43] Ahmad A., Saraswat D., El Gamal A., Johal G. (2021). CD&S Dataset: Handheld Imagery Dataset Acquired Under Field Conditions for Corn Disease Identification and Severity Estimation. arXiv.

[44] Ghose S. Corn or Maize Leaf Disease Dataset. https://www.kaggle.com/datasets/smaranjitghose/corn-or-maize-leaf-disease-dataset.

[45] Waema D., Mwangi W., Muriithi P. A Min-Max Based Data Normalization and Maximum Pooling Approach for Improved Maize Leaf Disease Detection. Proceedings of the 2025 IST-Africa Conference (IST-Africa).

[46] Isaev I., Dolenko S. (2018). Training with Noise Addition in Neural Network Solution of Inverse Problems: Procedures for Selection of the Optimal Network. Procedia Comput. Sci..

[47] Le Y., Yang X. (2015). Tiny ImageNet Visual Recognition Challenge. CS 231N.

